# C-to-G Base Editing Enhances Oleic Acid Production by Generating Novel Alleles of *FATTY ACID DESATURASE 2* in Plants

**DOI:** 10.3389/fpls.2021.748529

**Published:** 2021-10-26

**Authors:** Mid-Eum Park, Jae-Young Yun, Hyun Uk Kim

**Affiliations:** ^1^Department of Molecular Biology, Graduate School, Sejong University, Seoul, South Korea; ^2^Institutes of Green Bio Science and Technology, Seoul National University, Pyeongchang, South Korea; ^3^Department of Bioindustry and Bioresource Engineering, Plant Engineering Research Institute, Sejong University, Seoul, South Korea

**Keywords:** base editing, CRISPR, cytosine base editor, FAD2, oleic acid, vegetable oil

## Abstract

The demand for vegetable oil, which is mainly used for dietary purposes and cooking, is steadily increasing worldwide. It is often desirable to reduce unsaturation levels of fatty acids in order to increase storage stability and reduce *trans*-fat generation during cooking. Functional disruption of FATTY ACID DESATURASE 2 (FAD2) prevents the conversion of monounsaturated oleic acid to polyunsaturated linoleic acid, thereby enhancing the production of the desirable oleic acid. However, *FAD2* null alleles, due to growth defects under stress conditions, are impractical for agronomical purposes. Here, we aimed to attenuate FAD2 activity *in planta* while avoiding adverse growth effects by introducing amino-acid substitutions using CRISPR base editors. In *Arabidopsis*, we applied the adenine base editor (ABE) and cytosine base editor (CBE) to induce semi-random base substitutions within several selected *FAD2* coding regions. Isolation of base-edited *fad2* alleles with higher oleic acid revealed that the CBE application induced C-to-T and/or C-to-G base substitutions within the targeted sequences, resulting in an alteration of the FAD2 enzyme activities; for example, *fad2-144* with multiple C-to-G base substitutions showed less growth defects but with a significant increase in oleic acids by 3-fold higher than wild type. Our “proof-of-concept” approach suggests that equivalent alleles may be generated in vegetable oil crops *via* precision genome editing for practical cultivation. Our targeted semi-random strategy may serve as a new complementary platform for planta engineering of useful agronomic traits.

## Introduction

Vegetable oils contain three types of saturated fatty acids, palmitic acid (16:0), stearic acid (18:0), and arachidic acid (20:0) and five types of unsaturated fatty acids, oleic acid (18:1^Δ9^), linoleic acid (18:2^Δ9,12^), linolenic acid (18:3^Δ9,12,15^), 11-eicosenoic acid (20:1^Δ11^), and erucic acid (22:1^Δ13^). They can be used in industrial applications or as edible oils depending on their fatty acid composition. It is highly desirable to reduce the polyunsaturated fatty acid content and to increase the oleic acid (18:1^Δ9^), which is less vulnerable to oxidation (Holman and Elmer, 1947; Liu and White, 1992). FATTY ACID DESATURASE 2 (FAD2), which synthesizes 18:2^Δ9,12^ fatty acid from 18:1^Δ9^ fatty acid, was first identified as an endoplasmic reticulum (ER) membrane-bound protein (Okuley et al., 1994). The activity of FAD2 in plants is related to an increase in the content of dienoic fatty acids, hence increasing the resistance toward cold and salt stress, and is also known to affect development through salicylic acid (SA), abscisic acid, and jasmonic acid (JA) pathways (Martinez-Rivas et al., 2000; Kachroo et al., 2003; Regente et al., 2008).

Several attempts have been made to eliminate FAD2 function in plants in order to enhance desirable oleic acid production. In peanut and soybean, the *FAD2* was knocked out using transcription activator-like effector nucleases (TALENs; Haun et al., 2014; Wen et al., 2018). Likewise, disruption of the FAD2 function in peanut, soybean, and camelina has been demonstrated *via* clustered regularly interspaced short palindromic repeats (CRISPR)/CRISPR-associated protein 9 (Cas9; Jiang et al., 2017; Do et al., 2019; Yuan et al., 2019). However, *FAD2* null alleles are agronomically impractical because the loss of FAD2 function has adverse growth effects, resulting in a trade-off between reduction in yields and/or susceptibility to pathogens and increased oleic acid contents (Taylor et al., 2002; Anai et al., 2008; Pham et al., 2010). Of note, *fad2* null mutations in *Arabidopsis* have been reported to cause dwarf phenotypes at a lower ambient temperature (Miquel et al., 1993) whereas an EMS allele *fad2-1* appears to be more practical in terms of its growth phenotype (Lemieux et al., 1990). Targeting Induced Local Lesions in Genomes (TILLING) has been proposed as a useful approach for isolating point-mutant alleles for those genes, including *FAD2*, that lead to desirable agronomic traits and development as well (Lakhssassi et al., 2017).

Recently, derivatives of CRISPR/Cas9 systems have been developed to broaden the spectrum of CRISPR applications. One class of such derivatives is base editors (BEs). Nickase Cas9 (nCas9) fused to cytosine deaminases and to a laboratory-evolved adenine deaminase, referred to as a cytosine base editor (CBE; conversion of C to T) and an adenine base editor (ABE; conversion of A to G). These have been used to target deaminase domains to edit specific loci, without the need to generate double-strand breaks (DSBs), converting their respective nucleotides into other DNA bases (Komor et al., 2016; Nishida et al., 2016; Gaudelli et al., 2017). BEs can induce base substitutions leading to amino-acid change or splicing modifications. Two CBE systems, base editor 3 (BE3), and activation-induced cytidine deaminase (AID) adapt rAPOBEC1 from rat and PmCDA1 from sea lamprey as a cytidine deaminase domain, respectively (Komor et al., 2016; Nishida et al., 2016). CBEs often generate C-to-G substitutions as well *via* the BER (base excision repair) pathway, which served as a basis for the recent development of C-to-G base editors (Molla et al., 2020; Kurt et al., 2021; Zhao et al., 2021). Base editing has been applied to plants: The *acetyl CoA carboxylase* (*ACCase*) gene was modified in *A. thaliana* and *Oryza sativa* using enhanced ABE (Liu et al., 2020); Soybean flowering has been manipulated *via* amino-acid change within FLOWERING LOCUS T (FT), resulting from cytosine base editing (Cai et al., 2020); Watermelon and *Brassica napus* have been engineered to harbor herbicide resistance from the cytosine base editing in the *ACETOLACTATE SYNTHASE* (*ALS*; Tian et al., 2018; Wu et al., 2020).

In this study, we sought to generate *FAD2* missense alleles by implementing ABE or CBE within several target regions of the *FAD2* coding sequences in *Arabidopsis*. We selected those BE target sites for guide RNA (gRNA) design, based on the prediction of FAD2 protein structure, in order to generate genomic variations with base substitutions that are predicted to confer structural instability (Chavez Zobel et al., 2005; Li et al., 2020). For *Agrobacterium*-mediated plant transformation, we adapted the ABE binary vector, which has been reported in a previous study (Kang et al., 2018), and, for a CBE binary vector, we constructed a new pJY-RpAID vector based on plant-codon-optimized PmCDA fused to nCas9 under the control of *RPS5A* promoter. To isolate desirable *FAD2* alleles with increased oleic acid, we carefully investigated 26 T_1_ transformants by measuring the oleic acid contents through massive gas chromatography (GC) analyses on their corresponding T_2_ seeds. As a result, we obtained novel *FAD2* alleles showing significantly reduced desaturase activity, which led to enhanced oleic acid content. Genomic analyses confirmed that CBE implementation generated the desired novel missense alleles with multiple amino acid changes often resulting from the C-to-G rather than C-to-T substitutions. Our results also imply that our semi-random BE application to modify genes controlling important agronomic traits is a viable approach to produce novel designer alleles with new SNPs.

## Materials and Methods

### Plant Materials and Growth Conditions

All experiments were conducted using *Arabidopsis thaliana* Columbia-0 (Col-0) and *fad2-1* (Miquel and Browse, 1992) as an experimental control. *Arabidopsis* seeds were sterilized with 70% EtOH and 0.5% NaOCl and then were washed 7∼8 times with distilled water before sowing. The seeds were subject to stratification at 4°C for 3 days. They were then grown in 1/2 MS media containing 1% sucrose in a culture chamber at 23°C with a 16-h-light/8-h-dark cycle. About 14-day-grown seedlings in MS media were transferred to soil for further growth.

### Plasmid Construction

**FIGURE 1:**
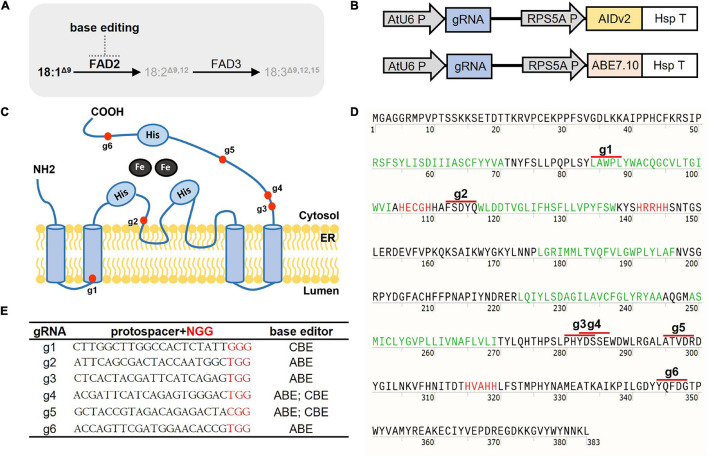
“Semi-random” base editing strategy for selected *FAD2* coding regions. **(A)** The function of FAD2 in desaturation of fatty acids and potential BE action in *Arabidopsis* seeds. FAD2 desaturates 18:1^Δ9^ to 18:2^Δ9,12^. FAD2 is subjected to base editing to alter its function. **(B)** Core structures of the CRISPR part in T-DNA from binary vectors harboring CBE and ABE in this study. gRNA expression is controlled by the U6 promoter, and AIDv2-dependent CBE and ABE7.10 are under the control of RPS5A promoters. **(C)** A schematic diagram of the FAD2 protein structure adapted from Zhang et al. (2012) ER-located FAD2 has six transmembrane domains and three histidine box motifs (His). Red dots denote BE target regions. **(D)** The FAD2 amino-acid sequence. *Arabidopsis* FAD2 is composed of 383 amino acids. Green-colored sequences indicate transmembrane domains, red-colored sequences indicate His motifs, and red lines indicate the potential amino acids that might be affected by BE targeting *via* corresponding gRNAs. **(E)** A list of selected gRNAs used for CBE and/or ABE with a protospacer sequence for *FAD2* editing in this study.

For basal CBE binary vector pJY-RpAID, plant (*A. thaliana*) codon-optimized coding sequences of PmCDA1 were synthesized by Integrated DNA Technology (Iowa, United States). First, pKI1.1R (Addgene #85808) (Shimada et al., 2010) was used to generate a pJY-RpEmpty vector, in which the original Cas9 sequences were removed, and two enzyme sites *Xma*JI and *Xho*I were inserted instead. Nickase Cas9 (D10A) and the codon-optimized AID were PCR amplified to generate pJY-RpAID binary vectors by the infusion cloning method (Takara, Japan) onto an *Xma*JI/*Xho*I-treated pJY-RpEmpty vector. The pJY-RpAID vector contains the following elements instead of the original Cas9 expression cassette from pKI1.1R: RPS5A promoter-SV40 NLS-nCas9 (D10A)-SV40 NLS-67aa linker-3X FLAG-PmCDA-SV40 NLS-UGI-heat shock protein 18.2 terminator (Figure 1B and Supplementary Figure 1). *Aar*I-mediated gRNA cloning was performed for plant transformation vectors as previously described (Kim et al., 2016). For base editing the *Arabidopsis FAD2* gene, six different 20-mer guide sequences (Figure 1E) were designed using the CRISPR RGEN Tool^1^ and cloned into *Aar*I-treated pJY-RpAID or a pJY-RpABE vector.

### Arabidopsis Transformation and Transgenic Plant Selection

*Agrobacterium* strain GV3101 was used to transform *A. thaliana via* the floral dipping method (Clough and Bent, 1998). The resulting T_1_ seeds were germinated and grown in 1/2 MS media containing 1% sucrose and 50 ng⋅μl^–1^ hygromycin. Only surviving plants in the media were selected and transferred to soil for further analyses. Leaf genomic DNA was extracted (Edwards et al., 1991) for Sanger sequencing and was analyzed whether base editing had occurred within the *FAD2* gene.

### Sanger Sequencing

The PCR was performed on extracted genomic DNA as a template using Ex Taq (Takara) to generate *FAD2* amplicons for subsequent sequencing analyses. A forward primer 5′-GCATTGTTTCAAACGCTCAA and a reverse primer 5′-TCATAACTTATTGTTGTACCAGTAC were used for 30-cycle PCR as following conditions: pre-denaturation (95°C for 5 min); denaturation (95°C for 30 s); annealing (52°C for 30 s); extension (72°C for 1 min); and post-extension (72°C for 10 min). A purification kit (Cosmo Genetech) was used to purify the PCR products. The quality and the quantity of the products had been determined using Nanodrop (Denovix) before the samples were deposited to a sequencing company (Bioneer).

### Fatty Acid Analysis

The 100 seeds were used for the gas chromatography (GC) analysis. About 500 μl of 5% sulfuric acid solution was dissolved in methanol, and toluene was added to each sample. To quantify the fatty acid contents, 15:0 fatty acid was dissolved in sulfuric acid. Each sample immersed with the solution was subject to the reaction in a water bath at 85°C for 2 h. Each sample was then added by 1 ml of 0.9% NaCl. After that, 1 ml of hexane was added to the solution three times and then was centrifuged at 330 × *g* for 2 min. The supernatant was aspirated, transferred to a 6-ml tube, purified fatty acid methyl ester (FAME) using a nitrogen concentrator (Eyela). The extracted FAME was dissolved in 200-μl hexane and then inserted into a GC vial. A DB-23 column (30 m × 0.25 mm, 0.25-μm film, Agilent) was used, and the extracted FAME was analyzed by GC-2030 (Shimadzu). The GC oven temperature was raised from 190 to 230°C at 3°C min^–1^.

### Germination Rate

The germination tests were conducted on the seeds that had been stratified for 3 days at 4°C. The germination events were scored when the roots started to appear in 1/2 MS media, 1/2 MS with 150-mM NaCl, and 1/2 MS with 300-mM Mannitol. All 1/2 MS used in the experiment contained 1% sucrose. The germination tests were carried out every 12 h for 4 days and replicated three times with 40 seeds per each line.

### Measurement of Root Growth

Col-0 and *fad2* alleles including *fad2-1* were grown under normal or stress conditions to monitor root development. For stress conditions, 4 DAG (days after germination) seedlings were transferred to 1/2 MS media, containing 75-mM NaCl or 200-mM Mannitol. The measurement of the root length was performed four times each for five individuals. The root length was measured on the 5th day of growing the seedling plant perpendicular to the light. The length measurement was carried out using the Image J program. Relative root length (%) was calculated as the average root length in stress MS media/average root length in MS media × 100.

### Statistical Analysis of the Data

The differences between the wild-type (Col-0) plants, *fad2-1* and the CBE lines, were identified by performing a one-way ANOVA using GraphPad Prism. Asterisks indicate significant differences compared to the wild-type plants (^∗^*p* < 0.05; ^∗∗^*p* < 0.01; ^∗∗∗^*p* < 0.001).

## Results

### Application of the Base Editors *in planta* to Generate “Attenuated” *fad2* Alleles

We aimed to generate potential “attenuated” *fad2* alleles with increased oleic acid at the cost of linoleic acid and linolenic acid, while minimizing adverse “trade-off” effects. We reasoned that certain C-to-T and/or A-to-G base substitutions, achieved by *in planta* CBE or ABE, within the coding regions of *FAD2* might attenuate, rather than abolish, their function (Figure 1A). To test this, we introduced random C-to-T and/or A-to-G base substitutions within several coding regions of *FAD2* locus *via in planta* CBE or ABE implementation. We first constructed a basal CBE binary vector pJY-RpAID in which AIDv2 is under the control of the RPS5A promoter, and the gRNA cloning cassette is under the control of the *Arabidopsis* U6 promoter (see section “Materials and Methods” for details; Figure 1B and Supplementary Figure 1). For the ABE binary vector, we adapted our pJY-RpABE vector reported previously (Figure 1B; Kang et al., 2018). Six distinct coding regions within the *FAD2* locus were selected as ABE and/or CBE targets based on the structural properties of the ER-membrane-bound FAD2 protein, which consists of six transmembrane domains and three histidine box motifs (Okuley et al., 1994; Shanklin et al., 1994; Figures 1C,D). We designed six independent gRNAs denoted as “g1–6” (Figure 1E) for BEs to target the selected coding regions, which correspond to the N-terminal transmembrane domain, tandem His-motif, a membrane-proximal cytosolic domain, cytosolic stretches, and C-terminal domains of the FAD2 protein (Figures 1C,D). Five ABE and three CBE constructs (Figure 1E) were created and transformed into *Arabidopsis* Col-0. Upon culture on selective media, 26 T_1_ plants were collected for analysis of BE transgenes and transferred to soil for later GC analyses of T_2_ seeds.

Individual pools of T_2_ seeds harvested from each T_1_ plant were subjected to parallel GC analyses to quantify their lipid compositions. Four independent T_1_ plants (named as g5CBE1-4) from the CBE constructs with g5CBE guide RNA showed increased oleic acid content in their T_2_ seeds (Supplementary Table 1). T_2_ seeds from g5CBE1, g5CBE2, g5CBE3, and g5CBE4 exhibited increased oleic acid contents up to 36.5, 36.2, 48.9, and 21.6%, respectively, while wild-type Col-0 and other transgenic lines (g1, g4CBEs and g2, g3, g4, g5, g6ABEs) oleic acid contents were no more than 20% (Supplementary Table 1; values with asterisks). Furthermore, g5CBE plants displayed decreased polyunsaturated fatty acid levels and increased eicosenoic acid (20:1^Δ11^) (Supplementary Table 1), implying that the desaturase activities of FAD2 protein had been compromised in those transgenic plants. We progressed to the next segregating T_2_ generation with g5CBE1 and g5CBE3, which had the highest amounts of oleic acid accumulation at their T_2_ seeds, in order to test whether *bona fide* cytosine base editing, which might be responsible for those increases, had occurred within the targeted *FAD2* region in those transgenic plants. Notably, other gRNAs transformants did not show any significant changes in fatty acids in the T1 generation, which may be due to low levels of either base editing or oleic acid changes. We decided to focus on g5CBE lines for the following experiments.

### Analyses of the Base-Editing Patterns and Lipid Contents From the *fad2* Lineages

**FIGURE 2:**
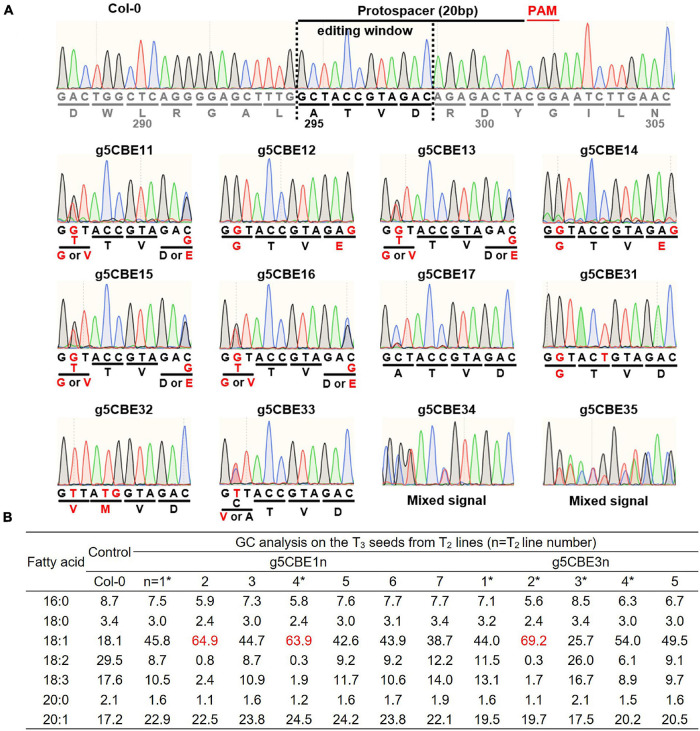
Characterization of the FAD2-targeting CBE-transgenic lines. **(A)** Chromatogram results from Sanger sequencing for *FAD2* genotyping within editing windows in the T_2_ plants. The editing window is specified in the upper Col-0 panel. Red letters indicate altered DNA and amino-acid sequences. **(B)** Analysis of fatty acid compositions of transgenic lineages. Red-colored numbers denote the three most increased oleic acid contents observed among T_2_ plants analyzed. Asterisks (*) indicate the representative T_2_ transgenic lines that were progressed to the next T_3_ generation for further characterization.

Seven (g5CBE11-17) and five T_2_ progenies (g5CBE31-35) from the g5CBE1 and the g5CBE3 T_1_ lineages, respectively, were analyzed for *FAD2* genotypes (Figure 2A and Supplementary Figure 2). Genomic DNA extracted from leaves of the T_2_ plants was subjected to Sanger sequencing covering the CBE target region of the *FAD2* gene (Figure 2A and Supplementary Figure 2). Interestingly, segregating C-to-G substitutions were observed at positions C2 and C12 in g5CBE11-16. Given that the protospacer adjacent motif (PAM) NGG sequences are regarded as the positions 21–23, these C-to-G substitutions are expected to result in the amino acid changes A295G and D298E, respectively (Figure 2A and Supplementary Figure 2). Among T_2_ plants, g5CBE12 and g5CBE14 appeared to have discrete C-to-G substitution chromatogram signals at the corresponding positions, indicating exclusive (A295G; D298E)-type amino acid changes had occurred (Figure 2A and Supplementary Figure 2). g5CBE11, g5CBE13, g5CBE15, and g5CBE16 showed mixed signals of C-to-G and C-to-T substitutions at the C2 position, and mixed signals of C and C-to-G at the C12 position (Figure 2A and Supplementary Figure 2). These results indicate that the edited bi-alleles at the C2 position and heterozygous C-to-G alleles at the C12 position had been transmitted to T_2_ plants. Of note, g5CBE17 showed all wild-type signals without any evidence of base editing events (Figure 2A and Supplementary Figure 2). Among g5CBE3 lineage, g5CBE31 plants also showed the A295G pattern from C-to-G conversion at the C2 position, accompanying with C-to-T conversion at C6 that would, anyway, result in synonymous mutation (Figure 2A and Supplementary Figure 2). In the g5CBE32, exclusive C-to-T substitutions occurred at the C2 position, resulting in A295V, and C-to-T and C-to-G at the C5 and C6 positions, respectively, resulting in T296M (Figure 2A and Supplementary Figure 2). In the g5CBE33 plant, heterozygous or mosaic C-to-T substitution at C2 was observed (Figure 2A and Supplementary Figure 2). The sequencing results for the g5CBE34 and g5CBE35 plants showed a mixed pool of sequencing traces, suggesting that larger genomic changes, such as indels, rather than point mutations might have occurred (Figure 2A and Supplementary Figure 2).

Gas chromatography analyses were then carried out on the corresponding T_3_ seeds from each T_2_ plant to investigate how the lipid composition had changed according to allelic differences with amino-acid changes. The oleic acid contents increased the most to 64.9, 63.9, and 69.2% in T_3_ seeds from g5CBE12, g5CBE14, and g5CBE32 plants, respectively, at the expense of the polyunsaturated fatty acids (Figure 2B; red-colored values). These lines are characterized by two concurrent amino acid changes at different residues resulting from two independent cytosine base-edited alleles (Figure 2A). The oleic acid content in T_3_ seeds from g5CBE11, g5CBE13, g5CBE15, and g5CBE16 showed moderate increases of oleic acid ranging from 42.6 to 45.8% (Figure 2A). Segregating C-to-T alleles at the C2 position and/or WT alleles at the C12 position might be responsible for those moderate increases in T_3_ seed oleic acid contents as compared to the maximum level increases from g5CBE12 and g5CBE14 (Figure 2B). g5CBE31 also showed an intermediate level of increase in oleic acid, indicating that A295G results in the partial suppression of FAD2 activity. g5CBE33, which contains heterozygous A295V, showed only slight increases in the oleic acid content to 25.7%, suggesting that A295V might affect FAD2 activity in a dosage-dependent manner (Figure 2B). g5CBE34 and g5CBE35 showed modest increases in oleic acid content up to 54% and 49.5%, respectively, but not to the maximum levels observed in g5CBE12 and g5CBE14 (Figures 2A,B).

### Isolation of the Base-Edited *fad2* Alleles

**FIGURE 3:**
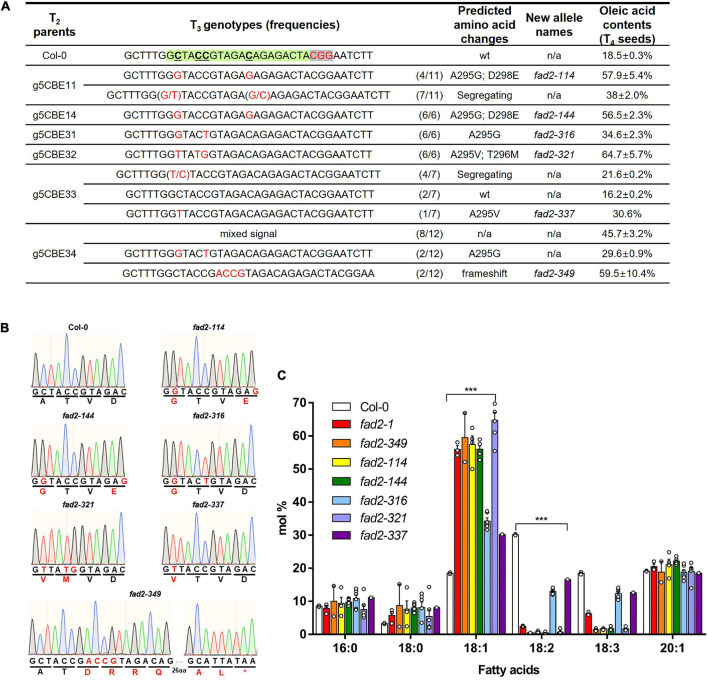
Characterization of the base-edited *fad2 alleles*. **(A)** A summary of T_3_ segregation patterns and analyses of oleic acid contents depending on each genotype. Green and gray letterboxes denote protospacer sequence and PAM sequence, respectively. Red letters indicate altered DNA sequences. According to fixed genotypes, novel *fad2* alleles were defined with predicted amino-acid changes. **(B)** Chromatogram results from Sanger sequencing for *FAD2* genotyping within editing windows in the defined *fad2* alleles. Red letters indicate altered DNA and amino-acid sequences. **(C)** Comparison of fatty acid compositions between T_4_ seeds from novel *fad2* alleles, *fad2-1*, and Col-0. A one-way ANOVA statistical analysis was used to identify differences between the Col-0 and *fad2* alleles (***p < 0.001). Values represent mean ± SD.

We proceeded with g5CBE11, g5CBE14, g5CBE31, g5CBE32, g5CBE33, and g5CBE34 to the next T_3_ generation in order to isolate homozygous alleles and to verify whether the changes in fatty acid composition depend on possible T_3_ segregations (Figures 2A,B; the line numbers with asterisks). g5CBE11 was selected for a representative among the T_2_ group containing biallelic A295G/A295V along with heterozygous D298E mutations. g5CBE14 and g5CBE32 were for A295G; D298E and A295V; and T296M allelic groups, respectively. g5CBE31 had the A295G mutation and a synonymous C-to-T mutation. g5CBE33 was chosen for the heterozygous A295V allele. g5CBE34 was also included in this progression group in anticipation of the isolation of an indel allele in their T3 generation. Sanger sequencing analyses were performed in the T_3_ progenies of each allelic group. As a result, we successfully isolated all possible base-edited T_3_ homozygous alleles for each genotype represented by g5CBE114 (A295G; D298E), g5CBE144 (A295G; D298E), g5CBE316 (A295G), g5CBE321 (A295V; T296M), and g5CBE337 (A295V), hereafter referred to as *fad2-114*, *fad2-144*, *fad2-316*, *fad2-321*, and *fad2-337*, respectively (Figures 3A,B and Supplementary Figure 3). For the T_3_ progenies of g5CBE34, we isolated a 4-bp insertional mutation allele, named *fad2-349* (frameshift mutation), presumably resulting in FAD2 knockout *via* a premature stop codon (Figures 3A,B and Supplementary Figure 3). Interestingly, segregating T_3_ progenies of g5CBE34 also present base-edited alleles (A295G), indicating that our CBE application had generated both base substitution and insertion mutations in a T_2_ g5CBE34 plant (Figure 3A and Supplementary Figure 3). The presence of a transgene was analyzed by PCR amplification of the nCas9 part for the T2 and T3 generations of g5CBE1 and g5CBE3 lines. The results indicate that the transgene had been segregated out from T3 in the g5CBE11 line and T2 in the g5CBE3 line (Supplementary Figure 4). Therefore, *de novo* additional base editing in the T3 generation was unlikely to have occurred.

Gas chromatography analyses carried out on all T_3_ genotypes revealed that all defined homozygous base-edited alleles showed significant increases in their oleic acid contents at their T_4_ seeds compared to wild-type Col-0 (Figures 3A,C and Supplementary Table 2). *fad2-321* (A295V; T296M) showed the highest increase, in oleic acid content, up to 64.7 ± 5.7%, among the base-edited *fad2* alleles (Figures 3A,C and Supplementary Table 2), which is consistent with observations in its parental T_2_ generation (Figure 2B). This increase is even higher than that of the previously reported *fad2-1* (A104T) (Lemieux et al., 1990) (55.9 ± 1.9% in our analyses, Figure 3C and Supplementary Table 2). *fad2-114* and *fad2-144* (A295G; D298E) showed enhanced oleic acid production, up to 57.9 ± 5.4% and 56.5 ± 2.3%, respectively (Figures 3A,C and Supplementary Table 2), more than their segregating siblings (38 ± 2%; Figure 3A and Supplementary Table 2). The increases are likely due to the homozygous changes in two amino acids at the same time, which had been segregating and thus having shown moderate increases in the previous generation g5CBE11 as well (Figure 2B). Particularly, the linoleic acid (18:2) and the linolenic acid (18:3) contents were dramatically decreased to below 2.4% and to below 2%, respectively, in both *fad2-321* and *fad2-114*, while those contents were 2.4 ± 0.2 and 6.2 ± 0.5% in *fad2-1* (Figure 3C and Supplementary Table 2), supporting the notion that the increases are due to “attenuated” desaturase activities of FAD2 in those alleles. Notably, the oleic acid content of the attenuated allele, such as *fad2-321*, was comparable to that of a *fad2-349* knockout allele in which the contents were measured as 59.5 ± 10.4% (Figures 3A,C and Supplementary Table 2).

### Characterization of the Growth Responses of *fad2* Alleles

**FIGURE 4:**
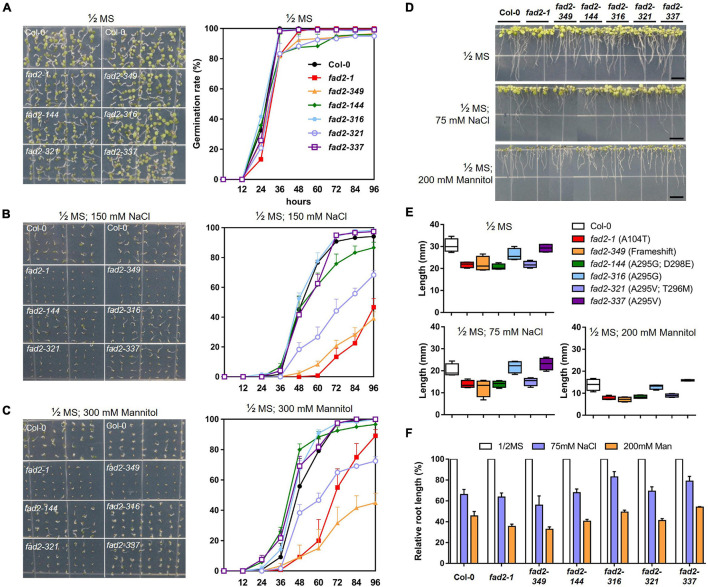
Analyses of physiological responses of “attenuated” *fad2* alleles. **(A–C)** Germination tests for *fad2* alleles conducted in **(A)**, 1/2 MS, **(B)**, 1/2 MS; 150-mM NaCl, and **(C)**, 1/2 MS; 300-mM Mannitol condition to test whether increases in oleic acid content influence germination under both normal and stress conditions. The pictures were taken after 96 h of sowing. Germination rates were measured every 12 h in triplicate. **(D–F)** Analyses of root growth rates of *fad2* alleles, whose seedlings had been grown for 4 days in 1/2 MS and then were transferred to both normal and stress conditions to allow root development for 5 days. The data presented in **(D)**, pictures taken to identify root lengths of 5-day-grown seedling under experimental conditions, **(E)**, graphs indicating the root lengths in different conditions, and **(F)**, a graph showing relative root lengths in 75-mM NaCl and 200-mM Mannitol when the root length in 1/2 MS was set to 100%. All values represent mean ± SD.

We hypothesized that certain “attenuated” *fad2* alleles in our study might not have as severe growth defects as the *fad2* knockout allele, and possibly even less severe than the previously reported *fad2-1* allele while maintaining increased oleic acid levels. In this regard, we decided to characterize our newly generated *fad2* alleles for phenotypical differences in terms of their growth responses. We analyzed two early growth responses, i.e., germination and root growth rate, for each allele under normal and stress conditions. We used experimental stress conditions of 150-mM NaCl and 300-mM Mannitol, and 75-mM NaCl and 200-mM Mannitol on top of 1/2 MS media for the germination and root growth test, respectively, with the intention of mimicking salt or water-deficit stress as has been described in previous studies (Zhang et al., 2012; Yu et al., 2017). For each of the alleles, germination tests were performed by scoring seed germination events at 12-h intervals during the initial 4 days after planting to assess whether increases in oleic acid content influence germination (Figures 4A–C). Under normal conditions, the germination frequencies of all tested alleles reached similar levels after 4 days, showing >95% germination success. Of note, *fad2-349*, *fad2-321*, and *fad2-144* alleles did show slightly reduced germination rates (∼72 h; Figure 4A), which coincide with relatively higher oleic acid contents observed in those alleles compared with others (Figure 3C). Upon both experimental stress conditions, *fad2-349* and *fad2-321* germination rates deteriorated sharply along with *fad2-1*, which showed significantly lower germination rates at all time points tested (Figures 4B,C). However, *fad2-144*, *fad2-316*, and *fad2-337* maintained germination rates comparable to that of WT (Figures 4B,C). These results suggest that those *fad2* alleles with increased oleic acid content also have enhanced resistance to stress susceptibility during germination in contrast to the case for *fad2-1*, *fad2-349*, and *fad2-321* (Figures 4B,C). Notably, *fad2-144*, which showed one of the highest levels of oleic acid content and contains two concurrent amino acid changes (A295G; D298E, Figure 3), was fairly resistant to the stress conditions tested for germination relative to *fad2-1* (Figures 4B,C).

Next, we assessed root growth under normal or stress conditions by measuring root lengths of 4-day-old seedlings from each of the *fad2* alleles (Figures 4D–F). Under normal growth conditions, all *fad2* alleles tested showed a range of the root length reduction compared to wild-type Col-0 (Figures 4D,E). For example, *fad2-144* and *fad2-321* showed considerable reduction similar to the reduction observed in *fad2-1* and knockout *fad2-349* (Figures 4D,E), suggesting that the corresponding allelic changes impede normal root development. On the other hand, *fad2-316* and *fad2-337* appeared to have insignificant levels of root reduction (Figures 4D,E), indicating that their allelic variation does not alter normal growth processes. Stress treatments caused a range of reduction rates of root lengths on the seedlings from each allele tested (Figures 4D,E). Under the salt-stress condition, the knockout allele *fad2-349* showed a reduction of root length to 55.8% of its length under the normal unstressed condition (Figure 4F), which is consistent with the reported growth defects in *FAD2* knockout mutations under salt stress. On the other hand, Col-0 and *fad2-1* showed 66% and 63.6% of their normal growth lengths, respectively (Figure 4F). Notably, *fad2-316* (A295G) and *fad2-337* (A295V), which display an intermediate increase of oleic acids from single amino-acid substitutions (Figure 3), showed less reduction in root length to 82.9% and 79.7%, respectively (Figure 4F). The result suggests that their growth might be even less affected by the salt stress condition than WT. *fad2-144* (A295G; D298E) and *fad2-321* (A295V; T296M), which were regarded as higher oleic acid-producing alleles with two concurrent amino-acid substitutions (Figure 3C), displayed 67.8 and 69.2% of relative root length under salt stress, similar to the reduction level observed in Col-0 (Figure 4F). The growth defects seemed more obvious in the water-deficit stress condition with 200-mM Mannitol, as the root length of *fad2-349* was reduced to 32.6% while Col-0 was to 45.5% (Figure 4F). Again, *fad2-316* and *fad2-337* presented 49.1 and 54% of relative root length, respectively, indicating significantly increased tolerance to water-deficit stress compared with knockout *fad2-349* (32.6%) or previously reported *fad2-1* (35.5%) (Figure 4F). The relative root lengths of *fad2-144* and *fad2-321* were 40.4 and 41.1%, respectively (Figure 4F). The result suggests that they are still more tolerant than *fad2-349* and *fad2-1* but have increased susceptibility to stress conditions comparable to Col-0.

Seed weight and fatty acid content of *fad2* alleles were measured (Supplementary Figure 5). Compared to wild-type Col-0, the weight of *fad2-1* was decreased, while the new *fad2* alleles showed an increase. The fatty acid content of the seeds showed some differences between the new *fad2* alleles. The fatty acid content per seed of *fad2-1* was not significantly different from that of wild type. The allele produced in this experiment had an increase in seed weight compared to the wild type, and there was no difference or a significant increase in the fatty acid content per seed. These results suggest that mutations in the *FAD2* gene can affect seed weight and fatty acid content. The correlation between *FAD2* gene mutation and fatty acid content needs further study.

## Discussion

**FIGURE 5:**
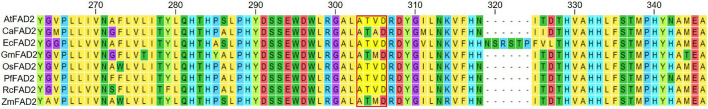
Alignment of FAD2 amino-acid sequences among *Arabidopsis* and various oil crops. Protein alignment was conducted by ClustalW in the MEGA7 program focusing on the g5 region in this study. The red box indicates the base editing window in this study. At, *Arabidopsis thaliana*; Ca, *Corylus avellana*; Ec, *Eucalyptus camaldulensis*; Gm, *Glycine max*; Os, *Oryza sativa*; Pf, *Physaria fendleri*; Rc, *Ricinus communis*; Zm, *Zea* mays. Different colors of amino acid are based on the polarity of the R group.

In our study, five different *fad2* alleles were isolated for increased oleic acid *via* semi-random targeting of BEs to six different genomic regions of the *FAD2* locus. All those higher oleic-acid alleles, interestingly, turned out to arise within the g5 gRNA targeting region, which represents the cytosolic stretch of ER-membrane-bound FAD2 protein. In principle, our laboratory-scale “pre-screen” may expedite the next process of recapitulating equivalent alleles in oil crops because one might focus on the corresponding g5 region to create such high oleic-acid variants *via* precision CBE targeting. Of note, the amino-acid residues harboring the allelic changes for increased oleic-acid content we have observed in our study are highly conserved in *FAD2* loci across major oil crops, implying direct value in applications (Figure 5). The A295V change observed in *fad2-321* and *fad2-337* has been reported to be one of the four combinatorial mutations conferring the competence in hydroxyl fatty acid production by manipulating the FAD2 function into FAH12-like property (Broun et al., 1998), supporting the notion that the corresponding residue may be pivotal to FAD2 activity. We reasonably anticipate that recapitulating suitable alleles such as a *fad2-144* equivalent in oil crops might be able to provide a clue to “agronomically applicable” genetic variants to mitigate adverse effects on plant growth and stress sensitivity that occurs in *FAD2* knockouts under certain growth conditions. Even intensive base editing within corresponding g5 regions in oil crops again with ABE and CBE might provide desired results with much less effort than traditional approaches.

We proposed and demonstrated that our targeted “semi-random mutagenesis” approach adapting CRISPR/BEs as a mutagenic source might be an effective alternative to generate novel *FAD2* alleles with desirable traits. All the reported *FAD2* mutations thus far were generated using conventional EMS, microRNA, and T-DNA approaches (Lemieux et al., 1990; Okuley et al., 1994; Song et al., 2010; Belide et al., 2012). The recent development of CRISPR/Cas9 facilitated the effective elimination of the FAD2 function in a targeted manner (Jiang et al., 2017; Do et al., 2019; Yuan et al., 2019; Bahariah et al., 2021). However, CRISPR/Cas9 induces DSBs and relies on the endogenous DNA repair process, i.e., NHEJ, to generate indels, which most frequently yield *FAD2* KO alleles, and thus make them unsuitable to field cultivation due to their growth defects. In this study, we aimed to combine the advantage of using CRISPR/BEs with a traditional “forward genetics” approach by randomly applying BEs to select genomic regions to induce missense mutations and then screen for candidate alleles with desirable phenotypes. Our “proof-of-concept” study reveals several advantages over conventional approaches, such as TILLING as follows: (1) much smaller size of screening population is required due to strategic targeting of mutagenic BEs within only select genomic regions; (2) a broader variety of point mutations can be induced, including not only base transitions, such as C-to-T and A-to-G but also C-to-G base transversions, some of which are not feasible using EMS mutagenesis; and (3) combinatorial *cis* mutations within a narrowly defined region can be obtained, as seen in *fad2-144* (A295G; D298E) and *fad2-321* (A295V; T296M). Furthermore, the results imply that our proposed “semi-random” mutagenic approach, particularly when combined with high-throughput gRNA library screening methods (Jae-Young et al., 2019), may offer a novel complementary platform for plant molecular breeding.

## Data Availability Statement

The original contributions presented in the study are included in the article/Supplementary Material, further inquiries can be directed to the corresponding authors.

## Author Contributions

M-EP, J-YY, and HUK performed the experiments, analyzed the data, and wrote the manuscript. All authors have read and approved the final manuscript.

## Conflict of Interest

The authors declare that the research was conducted in the absence of any commercial or financial relationships that could be construed as a potential conflict of interest.

## Publisher’s Note

All claims expressed in this article are solely those of the authors and do not necessarily represent those of their affiliated organizations, or those of the publisher, the editors and the reviewers. Any product that may be evaluated in this article, or claim that may be made by its manufacturer, is not guaranteed or endorsed by the publisher.

## Footnotes

1
http://www.rgenome.net
