# Supplementary material for: C-to-G Base Editing Enhances Oleic Acid Production by Generating Novel Alleles of FATTY ACID DESATURASE 2 in Plants
Source: Front Plant Sci. 2021 Oct 26;12:748529. doi: 10.3389/fpls.2021.748529 (PMC8576475; doi:10.3389/fpls.2021.748529)
Supplement: Supplementary file 2 [file Presentation_1.pptx]

## Slide 1
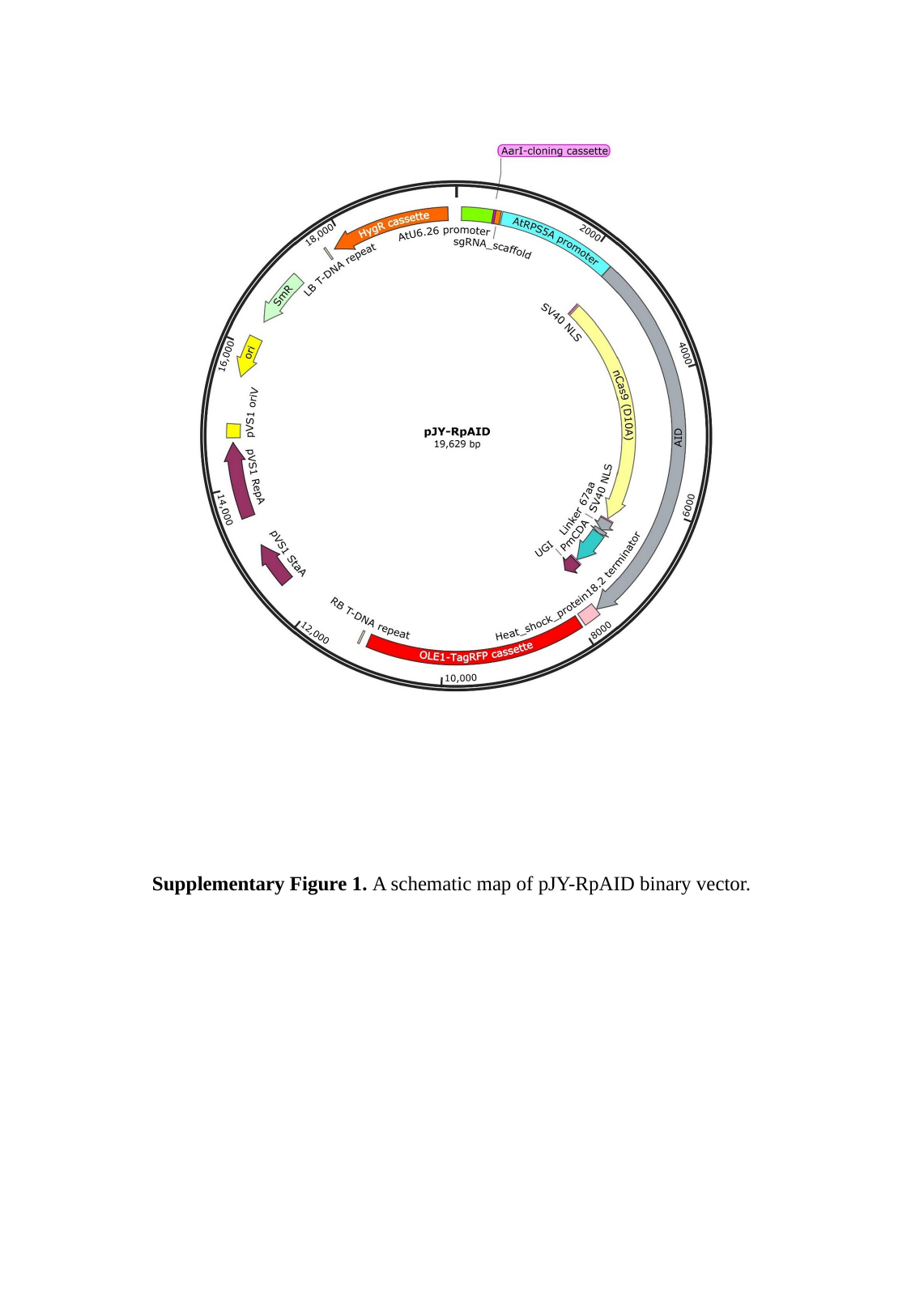

Supplementary Figure 1. A schematic map of pJY-RpAID binary vector.

## Slide 2
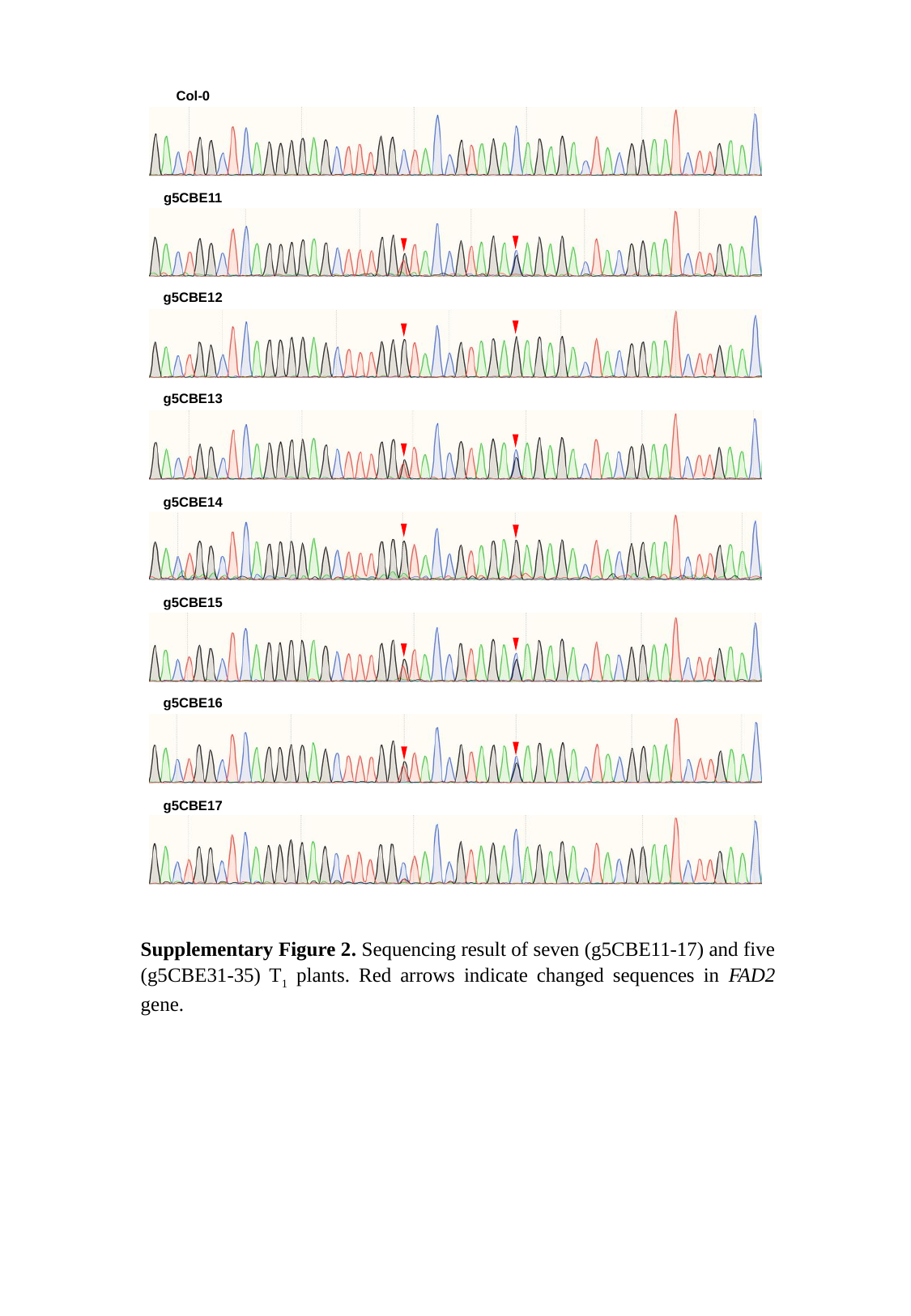

Col-0
g5CBE11
g5CBE12
g5CBE13
g5CBE14
g5CBE15
g5CBE16
g5CBE17
Supplementary Figure 2. Sequencing result of seven (g5CBE11-17) and five (g5CBE31-35) T1 plants. Red arrows indicate changed sequences in FAD2 gene.

## Slide 3
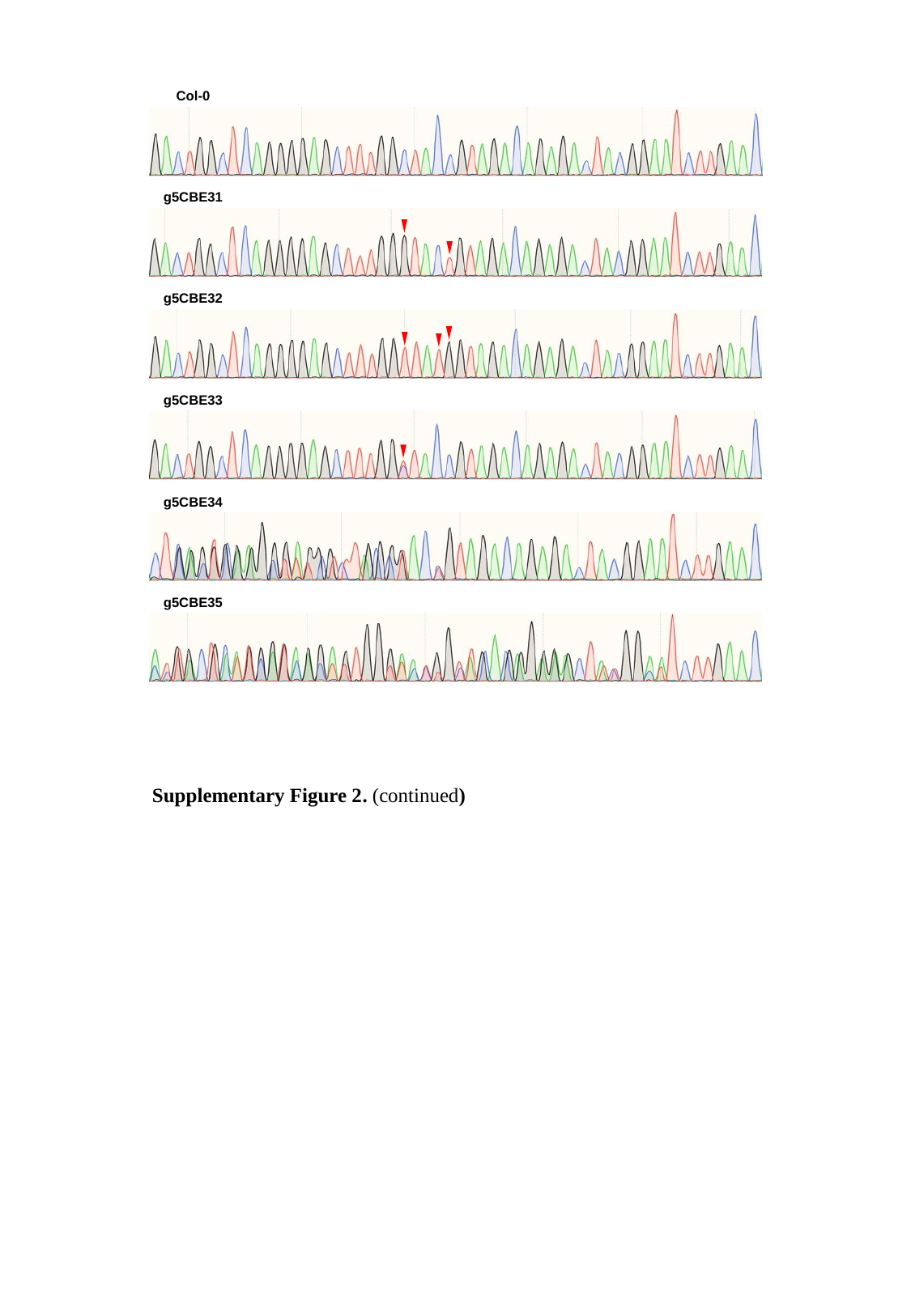

Col-0
g5CBE31
g5CBE32
g5CBE33
g5CBE34
g5CBE35
Supplementary Figure 2. (continued)

## Slide 4
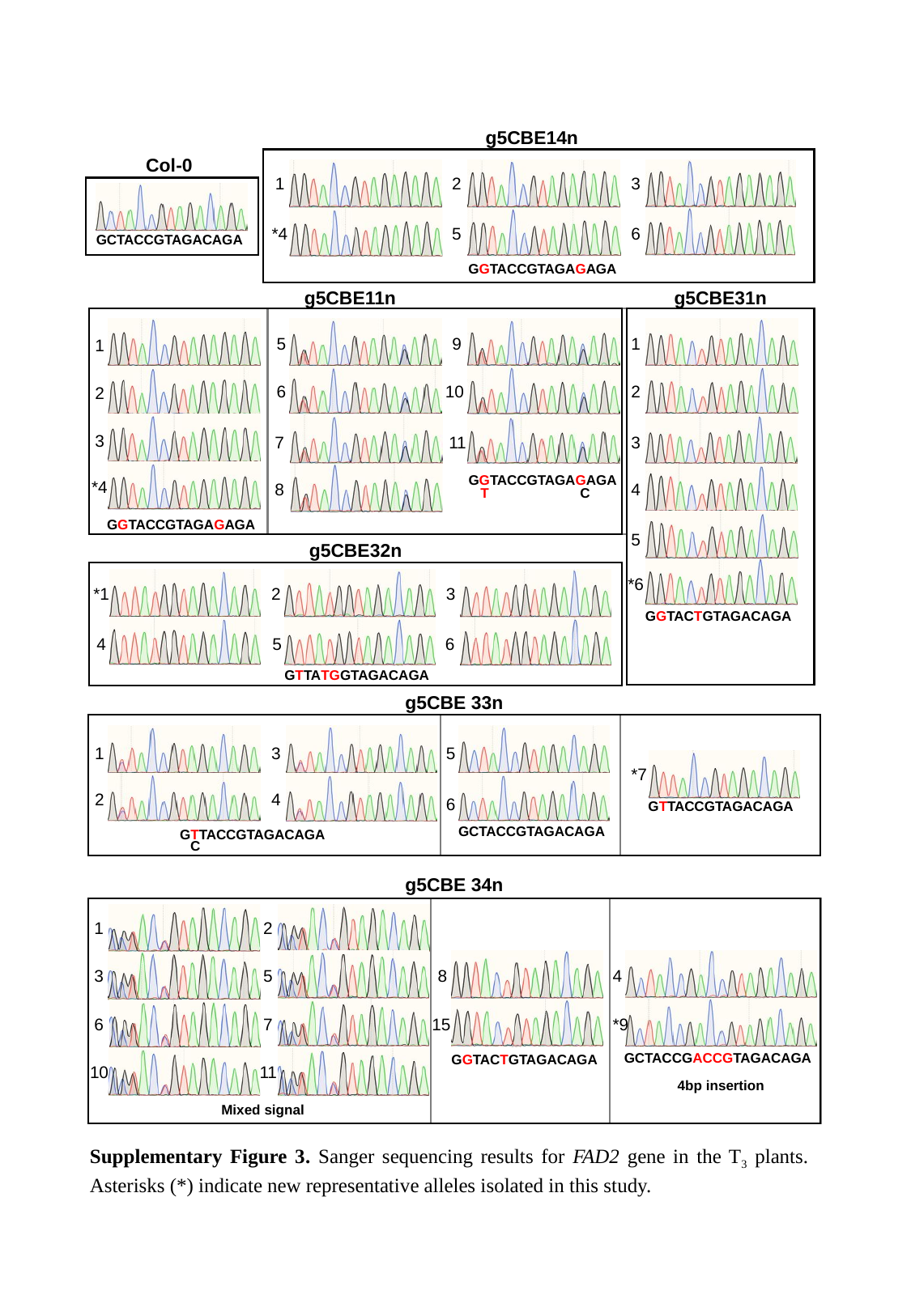

g5CBE14n
Col-0
1
2
3
*4
5
6
GCTACCGTAGACAGA
GGTACCGTAGAGAGA
g5CBE11n
g5CBE31n
5
9
1
1
6
10
2
2
3
7
11
3
GGTACCGTAGAGAGA
*4
8
4
T
C
GGTACCGTAGAGAGA
5
g5CBE32n
*6
*1
2
3
GGTACTGTAGACAGA
4
5
6
GTTATGGTAGACAGA
g5CBE 33n
1
3
5
*7
2
4
6
GTTACCGTAGACAGA
GCTACCGTAGACAGA
GTTACCGTAGACAGA
C
g5CBE 34n
1
2
3
5
8
4
6
7
15
*9
GCTACCGACCGTAGACAGA
GGTACTGTAGACAGA
10
11
4bp insertion
Mixed signal
Supplementary Figure 3. Sanger sequencing results for FAD2 gene in the T3 plants. Asterisks (*) indicate new representative alleles isolated in this study.

## Slide 5
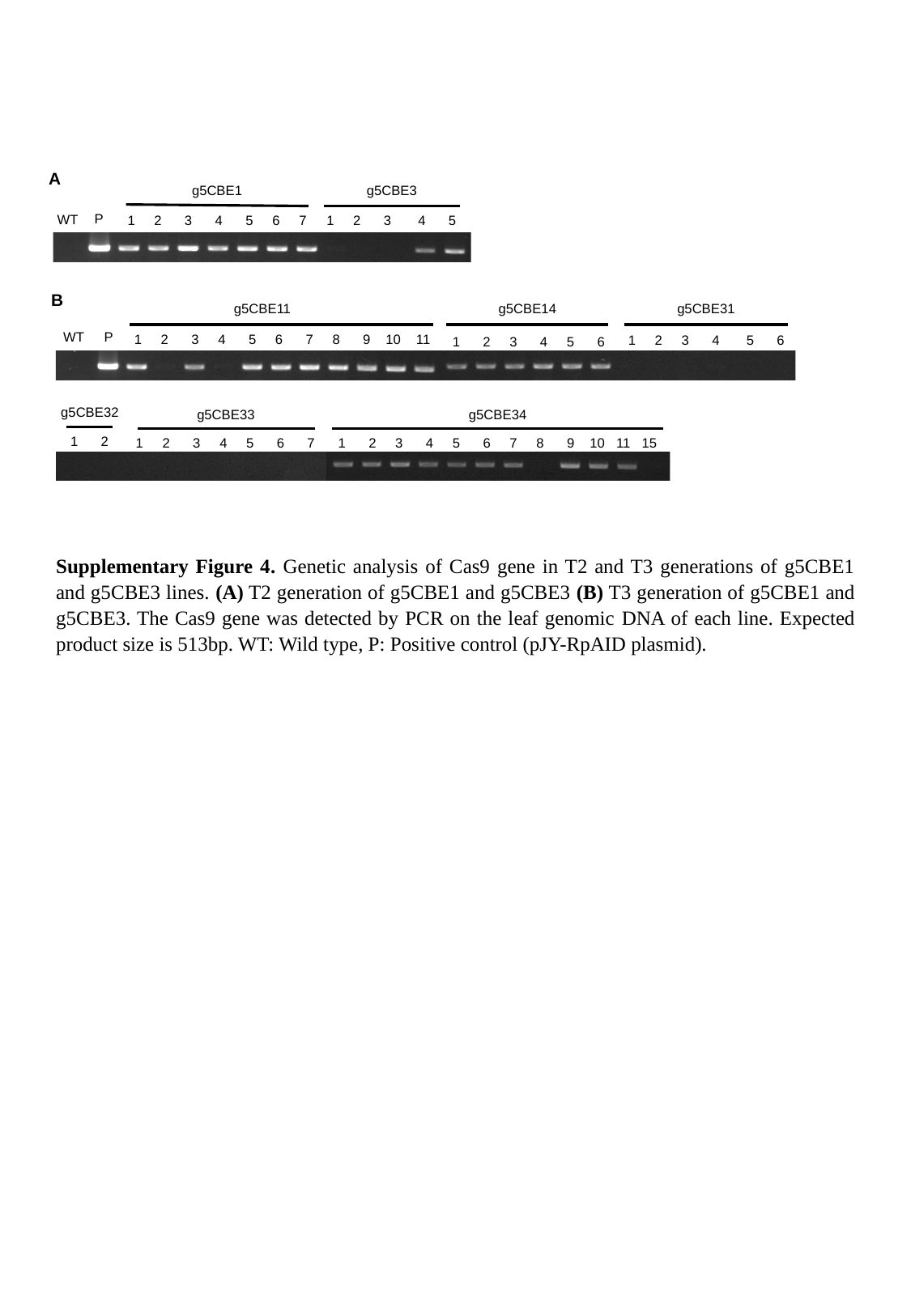

A
g5CBE1
g5CBE3
P
WT
1 2 3 4 5 6 7
1 2 3 4 5
B
g5CBE11
g5CBE14
g5CBE31
WT
P
1 2 3 4 5 6 7 8 9 10 11
1 2 3 4 5 6
g5CBE32
g5CBE33
g5CBE34
1 2
1 2 3 4 5 6 7
1 2 3 4 5 6 7 8 9 10 11 15
1 2 3 4 5 6
Supplementary Figure 4. Genetic analysis of Cas9 gene in T2 and T3 generations of g5CBE1 and g5CBE3 lines. (A) T2 generation of g5CBE1 and g5CBE3 (B) T3 generation of g5CBE1 and g5CBE3. The Cas9 gene was detected by PCR on the leaf genomic DNA of each line. Expected product size is 513bp. WT: Wild type, P: Positive control (pJY-RpAID plasmid).

## Slide 6
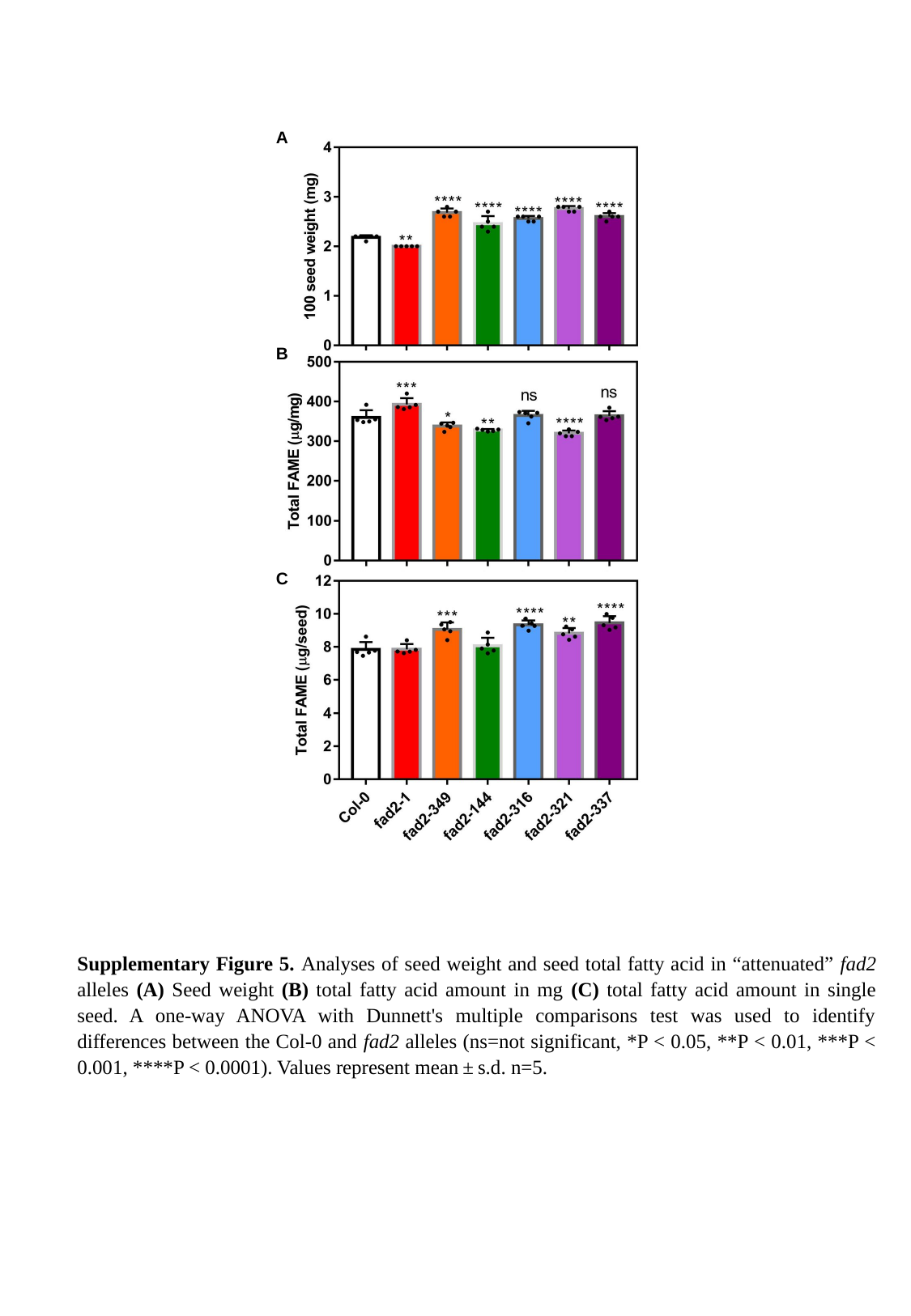

A
B
C
Supplementary Figure 5. Analyses of seed weight and seed total fatty acid in “attenuated” fad2 alleles (A) Seed weight (B) total fatty acid amount in mg (C) total fatty acid amount in single seed. A one-way ANOVA with Dunnett's multiple comparisons test was used to identify differences between the Col-0 and fad2 alleles (ns=not significant, *P < 0.05, **P < 0.01, ***P < 0.001, ****P < 0.0001). Values represent mean ± s.d. n=5.
